# Δ*lpp* mutant *E. coli* reduces lipid content in *Caenorhabditis elegans* via phosphatidylglycerol-mediated inhibition of fatty acid biosynthesis

**DOI:** 10.1128/msystems.00155-26

**Published:** 2026-06-17

**Authors:** Dianshuang Zhou, Liwei Tang, Mengdie Ni, Wen Yang, Xiangming Wang, Ying Li, Zuobin Zhu

**Affiliations:** 1 Jiangsu Engineering Center for Precision Diagnosis and Treatment Research of Polygenic Diseases, Key Laboratory of Genetic Foundation and Clinical Application, Department of Genetics, Xuzhou Medical University 38044 , Xuzhou, China; 2 The First People’s Hospital of Lianyungang, Lianyungang, China; 3 Department of Cell Biology, Laboratory for Clinical Medicine, School of Basic Medical Sciences, Capital Medical University 12517, Beijing, China; 4 Medical Technology College, Xuzhou Medical University, Xuzhou, China; Wageningen University, Wageningen, Netherlands; University Medical Center Groningen, Groningen, Netherlands

**Keywords:** mutant *E. coli*, lipid, phosphatidylglycerol, *Caenorhabditis elegans*

## Abstract

Although the gut microbiota is known to influence host metabolism, how specific bacterial genetic mutations modulate lipid metabolism remains unclear. In this study, we integrated *in vivo* lipid visualization, whole-transcriptome RNA sequencing, and lipidomics to investigate the effects of the Δ*lpp* mutant of *Escherichia coli* on *Caenorhabditis elegans* (*C. elegans*). Our results demonstrated that the Δ*lpp* mutant significantly reduced fat content across three *C. elegans* strains. Transcriptome analysis revealed substantial alterations in the expression of genes associated with fatty acid metabolism, particularly those involved in fatty acid biosynthesis, which were notably downregulated. Lipidomic profiling confirmed an upregulation of phosphatidylglycerol (PG) and indicated its potential involvement in regulating fatty acid biosynthesis. Importantly, PG supplementation significantly decreased lipid accumulation in *C. elegans*. Furthermore, we identified and validated four downregulated genes whose reduced expression was associated with lower lipid levels. These findings underscore the therapeutic potential of engineered probiotics in lipid-related metabolic disorders and offer novel mechanistic insights into how genetic variations in gut microbes influence host metabolic health.

**IMPORTANCE:**

Our findings carry several potential implications: they not only reveal a novel pathway through which a bacterial mutant affects host lipid metabolism via phosphatidylglycerol-mediated inhibition of fatty acid biosynthesis but also underscore the potential of engineered microbial strains as precision probiotics for the treatment of lipid-associated disorders.

## OBSERVATION

The complex interactions between microorganisms and their hosts are crucial to numerous physiological processes in multicellular organisms. Gut microbiota dysbiosis perturbs host metabolic and immune networks (1, 2). Lipid metabolism is a dynamic equilibrium process involving the synthesis, absorption, catabolism, and transportation of lipids in organisms. Its dysregulation is closely associated with multiple major diseases, including cardiovascular disorders, obesity, diabetes mellitus, and neurodegenerative diseases (3–5). Recent studies indicate that microorganisms can influence the host’s lipid metabolism, and probiotic intervention can improve lipid metabolism disorders (6, 7). For example, supplementation with *Bacteroides vulgatus* alleviates high-fat diet-induced obesity and metabolic dysfunction (8). Previous studies have demonstrated that genetic variations in microorganisms, including gene presence/absence, structural variations, and copy number alterations, are closely associated with host physiology and disease phenotypes. Obesity is associated with a higher copy number of thioredoxin 1 (K03671) in the genus *Clostridium* (9). A recent study further reveals that bacterial structural variations, particularly those involved in ion and amino acid metabolism as well as bacterial growth regulation, are significantly associated with autism spectrum disorder and hold promise as diagnostic biomarkers (10). Our previous research demonstrated that *S. cerevisiae* mutant strains can regulate the lifespan, sleep patterns, and locomotor performance of *Drosophila melanogaster* (11). In this study, we found that the Δ*lpp* mutant *Escherichia coli*, which lacks a gene encoding the major outer membrane lipoprotein *Lpp* (a key structural component covalently linking the outer membrane to peptidoglycan) (12), significantly reduced the lipid content in *Caenorhabditis elegans*, and its metabolite phosphatidylglycerol (PG) was a key molecule for this effect.

Body fat in *C. elegans* is predominantly stored in the intestinal tract and epidermis-like cells (13). Based on the Kyoto Encyclopedia of Genes and Genomes (KEGG) database, functional pathways associated with lipid metabolism were screened, and genes within these pathways were extracted. Genes interacting with those in lipid metabolism–related pathways (interaction score > 900) were obtained using the STRING database. By matching against the *E. coli* genome-wide knockout library, 133 mutant strains with corresponding gene disruptions were acquired. These mutant strains were individually fed to *C.elegans* strain N2, with the wild-type strain BW25113 as the control. Primary screening was performed via Oil Red O staining, with *P* < 0.05 set as the significance threshold, and each experiment was repeated three times. A total of 44 candidate mutants were identified from the primary screen and further validated in two *C. elegans* strains, LIU1 and LIU2, which carry fluorescent lipid droplet markers (Table S1). After comprehensively evaluating the lipid-lowering effects of each mutant across the three *C. elegans* strains, the Δ*lpp* mutant was ultimately selected for subsequent studies.

**Fig 1:**
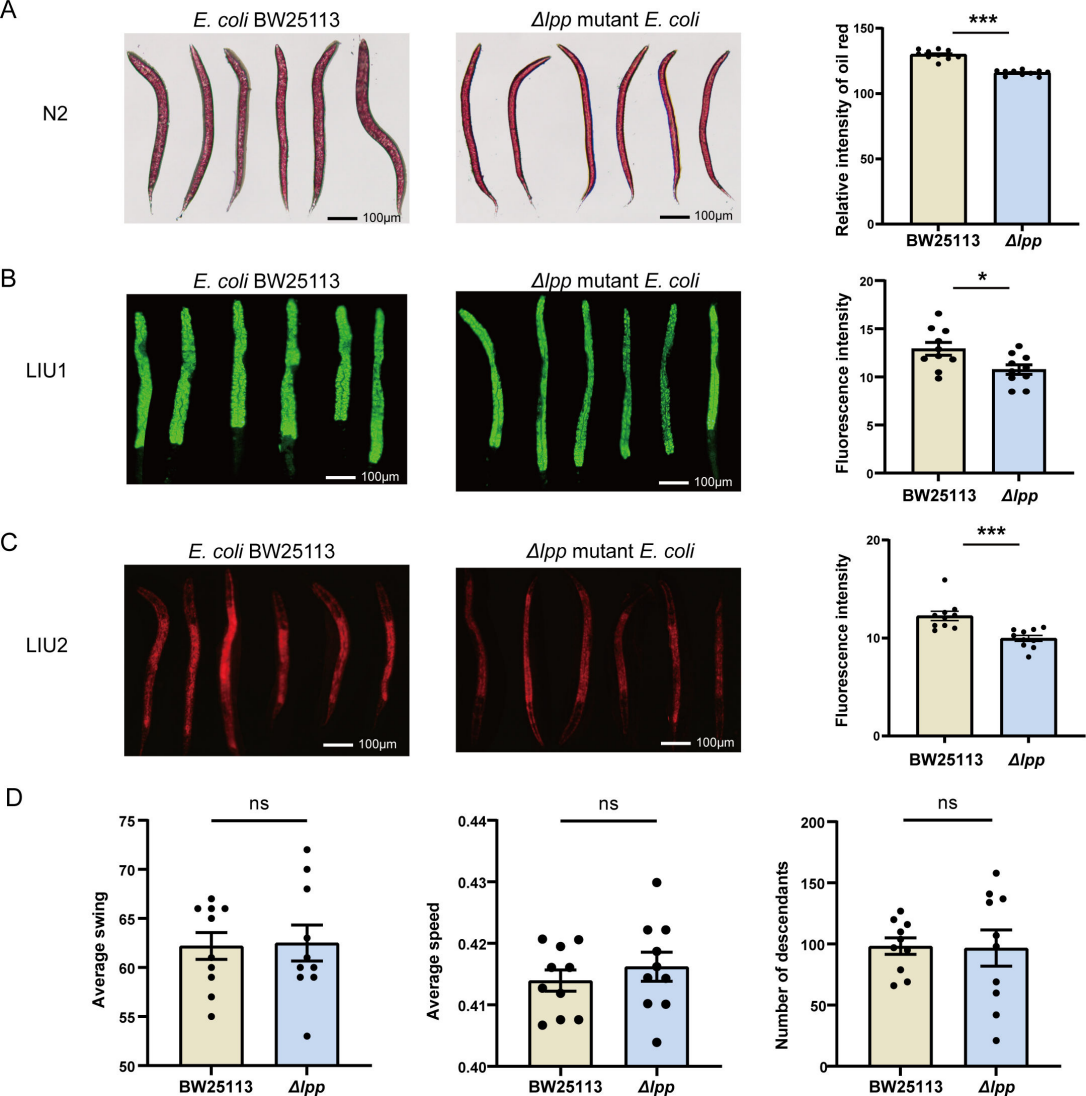
Δ*lpp* mutant can significantly reduce the fat content of *C. elegans*. (**A–C**) Oil Red O staining of N2 nematodes fed with BW25113 and Δ*lpp* mutant and fluorescence imaging of LIU1 and LIU2 nematodes at Day 1 post-L4 stage. The bar chart data are the mean ± standard error of three biological replicates (*P < 0.05, ***P < 0.001). (**D**) Comparison of locomotor ability and number of descendants of N2 nematodes fed with BW25113 and Δ*lpp* mutant (ns represents *P* > 0.05).

Compared to the wild-type control *E. coli* BW25113, exposure to the Δ*lpp* mutant led to a significant reduction in Oil Red O staining intensity by 10.7% (*P* < 0.001) in N2, while fluorescent intensity declined by 16.8% (*P* < 0.05) in LIU1 and 18.7% (*P* < 0.001) in LIU2 (Fig. 1A through C; Table S2). To control for potential variations in body size, lipid content was normalized to unit surface area, revealing that the mutant significantly reduced lipid levels without altering body size (Fig. S1). Additionally, we evaluated reproductive capacity and locomotor activity to exclude the possibility of nonspecific physiological effects. No significant differences were detected between the control and Δ*lpp*-fed groups (Fig. 1D), suggesting that the observed reduction in fat content reflects a specific metabolic effect rather than a general physiological impairment.

**Fig 2:**
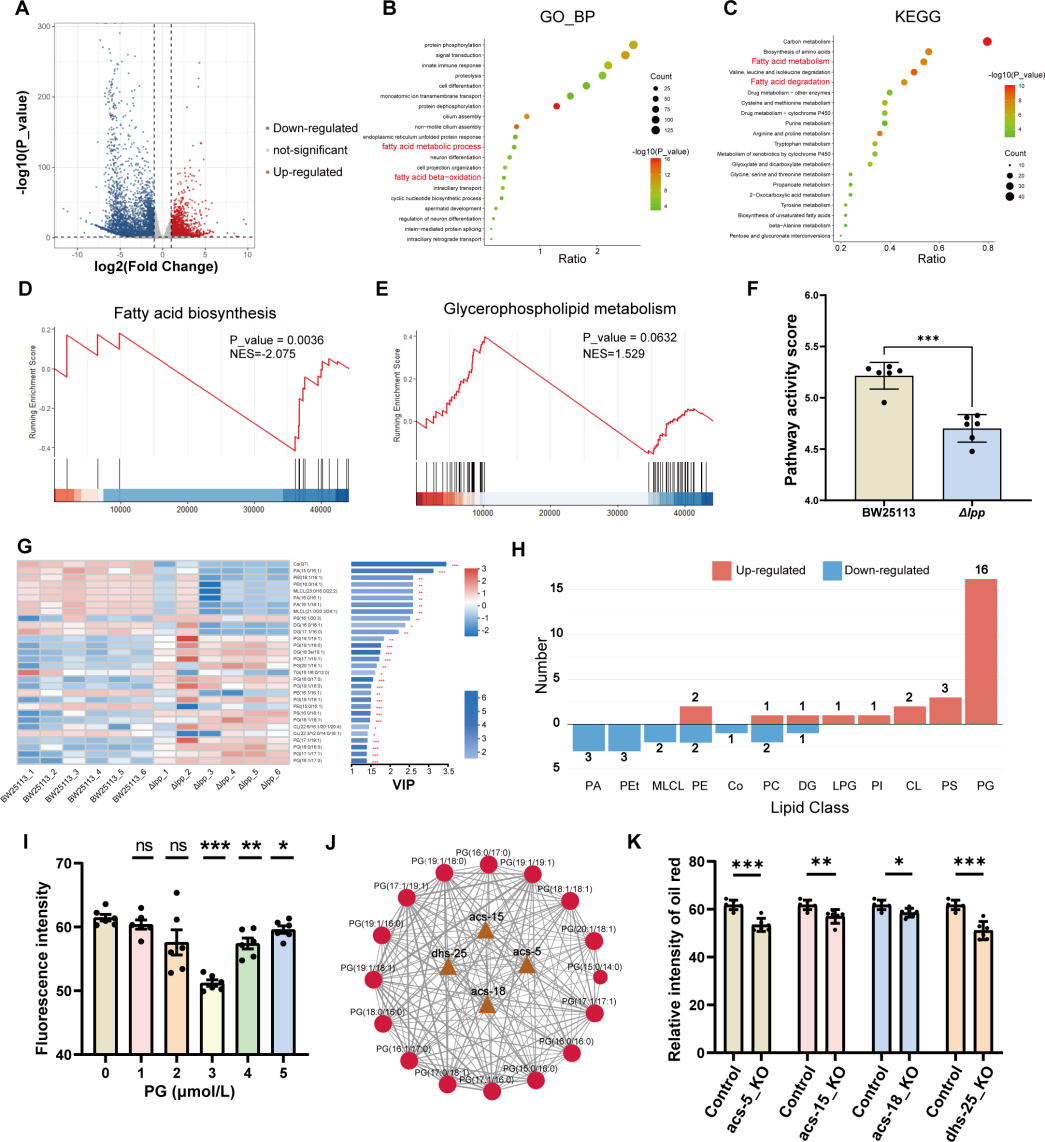
The metabolic product PG of Δ*lpp* mutant *E. coli* can reduce the synthesis of fatty acids in *C. elegans*. (**A**) Volcano map of differentially expressed genes in *C. elegans* fed with Δ*lpp* mutant and wild-type BW25113 *E. coli*. (**B** and **C**) Gene Ontology (GO) and KEGG enrichment analyses of differentially expressed genes. (**D**) Fatty acid biosynthesis pathway was a significantly enriched downregulated pathway based on GSEA. (**E**) Glycerophospholipid metabolism pathway was a marginally significant upregulated pathway based on GSEA. (**F**) The violin chart showed the activity score of the fatty acid biosynthesis pathway for the Δ*lpp* and BW25113 groups. *** represents *P* < 0.001. (**G**) Metabolic levels and VIP values of the top 30 significantly different lipid metabolites. (*P < 0.05, **P < 0.01, ***P < 0.001, ns represents *P* > 0.05). (**H**) The number of differential metabolites in the Δ*lpp* group. (**I**) The reduction in N2 nematodes’ lipid content following supplementation with different concentrations of PG. (**J**) The four nematode genes significantly associated with PG identified using the Pearson correlation coefficient. (**K**) Comparison of fat content between four gene knockout *C. elegans* strains and the control.

To elucidate the molecular mechanisms underlying this phenotype, we conducted whole-transcriptome sequencing on N2 nematodes fed with either the Δ*lpp* mutant or the wild-type control BW25113 *E. coli*. Gene expression analysis demonstrated high intra-group reproducibility (Fig. S2A). A total of 5,441 differentially expressed genes were identified, including 2,351 upregulated and 3,090 downregulated genes (Fig. 2A). Functional enrichment analysis using DAVID revealed significant enrichment in biological processes such as signal transduction, protein phosphorylation, fatty acid β-oxidation, and fatty acid metabolism (Fig. 2B). KEGG and Reactome pathway analyses further highlighted enrichment in metabolic pathways, particularly those involved in amino acid and fatty acid metabolism (Fig. 2C; Fig. S2B). Gene Set Enrichment Analysis (GSEA) specifically indicated a downregulation of the fatty acid biosynthesis pathway (*P* = 0.0037, false discovery rate (FDR) = 0.011, normalized enrichment score [NES] = −2.075) (Fig. 2D), whereas glycerophospholipid metabolism showed a marginal upregulation (Fig. 2E). Single-sample GSEA confirmed that fatty acid biosynthesis activity was significantly suppressed in the *Δlpp* group (Fig. 2F), suggesting that the mutant inhibits host fatty acid synthesis.

To elucidate the metabolic intermediates responsible for Δ*lpp* mutant *E. coli*-induced inhibition of fatty acid biosynthesis in *C. elegans*, we performed lipidomic profiling on Δ*lpp* mutant *E. coli* and the wild-type strain BW25113. The lipidomics sequencing of the samples was carried out using a liquid chromatography-mass spectrometry analysis platform. A total of 314 lipid metabolites were detected, among which 131 lipid metabolites were detected in the positive ion mode and 183 lipid metabolites were detected in the negative ion mode. These metabolites belong to four categories (fatty acids, glycerolipids, glycerophospholipids, and sphingolipids) and 30 subclasses (Fig. S3). We first used orthogonal partial least squares discriminant analysis to establish a relationship model between levels of metabolites and differences of samples. The results showed that intra-group reproducibility was consistent and samples were clustered together. At the same time, there were apparent differences between groups that formed separate clusters (Fig. S4). Based on the criteria of variable importance in projection (VIP) > 1 and FDR < 0.05 for differential metabolites, we identified 27 upregulated and 13 downregulated differential metabolites (Fig. 2G). We performed statistical analysis of differential metabolites based on lipid subclasses and found that the PG class was widely upregulated in the Δ*lpp* group. A total of 16 metabolites belonging to PG were significantly upregulated in the Δ*lpp* group, with no significant downregulation observed. In contrast to PG, phosphatidylethanolamine, phosphatidic acid, and giacylglycerol exhibited both upregulation and downregulation (Fig. 2H). The alterations in these lipid species, which are key lipid molecules in phospholipid metabolism, suggested a broader remodeling of the lipid metabolic network in the Δ*lpp* mutant. The quantification of Oil Red O staining intensity of N2 nematodes was significantly reduced by 16.8% (*P* < 0.001) after the supplement of 3 μM PG (Fig. 2I). Consistent results were also obtained when PG was supplemented in LIU1 nematodes (Fig. S5). Therefore, we concluded that Δ*lpp* mutant *E. coli* upregulated the production of PG, and PG was a key factor influencing fatty acid biosynthesis in *C. elegans*.

Since lipids can modulate gene expression, we performed correlation analysis between PG levels and host transcriptomic changes by ggClusterNet2 (14). We identified four genes (*dhs-25*, *acs-5*, *acs-15*, and *acs-18*) exhibiting strong negative correlation with PG (*r* < −0.8, *P* < 0.05) (15) (Fig. 2J). These genes, which are involved in fatty acid biosynthesis, were downregulated in the Δ*lpp* mutant group. Quantitative polymerase chain reaction assays validated their differential expression patterns (Fig. S6). Meanwhile, consistent results were also observed in *C. elegans* supplemented with PG (Fig. S7), indicating that PG regulates these genes either directly or indirectly. To further confirm the functional relevance of these downregulated genes in lipid metabolism, we used knockout strains and assessed their total lipid content. Compared with the wild-type strain, all four knockout strains exhibited significantly reduced fat accumulation, with decreases of 13.1%, 7.7%, 5.3%, and 17.4%, respectively (Fig. 2K). Consistent with these phenotypic observations, previous studies and functional annotations support the roles of these genes in lipid homeostasis: DHS-25 is an ortholog of human 17β-HSD8, while DHS-3 (short-chain dehydrogenase/reductase) localizes to lipid droplets, and its deletion has been shown to induce lipid depletion in model organisms (16, 17). Additionally, the *acs* family genes encode acyl-CoA synthetases, and impairment of *acs-1* specifically reduces branched-chain fatty acid levels and disrupts lipid droplet morphology—key features of altered lipid metabolism (18). Collectively, these findings support the hypothesis that the four genes we identified play essential roles in regulating lipid metabolism in *C. elegans*.

In summary, this study demonstrates that the Δ*lpp* mutant of *E. coli* significantly reduces fat content in *C. elegans* through transcriptomic and lipidomic reprogramming, with PG acting as a key microbial metabolite that suppresses fatty acid synthesis. PG is a crucial precursor in the biosynthesis of bacterial membrane phospholipids (19). Braun’s lipoprotein encoded by the *lpp* gene is the sole molecule that covalently links the outer membrane to peptidoglycan in *E. coli* (20). Its deletion compromises outer membrane integrity and increases cell membrane permeability (21, 22). Although *lpp* deficiency does not significantly affect the basic growth of *E. coli* (21), outer membrane stress may trigger the reprogramming of bacterial membrane lipid metabolism. In the present study, the Δ*lpp* mutation resulted in a significant upregulation of PG levels, which most likely arises from bacterial cell envelope stress and adaptive remodeling induced by *lpp* deletion. Notably, the Δ*lpp* mutant has also been shown to extend the lifespan of *C. elegans* (23), suggesting a potential link between the regulation of lipid metabolism and longevity. This study found that the Δ*lpp* mutant inhibits fatty acid biosynthesis and fat accumulation in the host by increasing the production of PG and suggests that regulating the export of specific metabolites by the gut microbiota may represent a potential strategy for modulating host metabolism.

## Supplementary Material

Reviewer comments

## Data Availability

The gene expression profile of *C. elegans* and the lipidomics data of *E. coli* are provided in Table S3. RNA-Seq data have been deposited to the GEO database with accession no. GSE331223. All raw data, analytical scripts, and detailed experimental protocols are made available to enable other researchers to fully replicate and validate the reported results.

## SUPPLEMENTAL MATERIAL

Supplemental text msystems.00155-26-s0001.docx Extended methods.

Supplemental text msystems.00155-26-s0002.docx Fig. S1 to S7.

Table S1 msystems.00155-26-s0003.xlsx Significance analysis of *E. coli* mutants reducing lipid content in *C. elegans*.

Table S2 msystems.00155-26-s0004.xlsx Staining intensities of three strains of nematodes.

Table S3 msystems.00155-26-s0005.xlsx Gene expression profile of *C. elegans* and lipidomics data.

OPEN PEER REVIEW reviewer-comments.pdf An accounting of the reviewer comments and feedback.

ASM does not own the copyrights to Supplemental Material that may be linked to, or accessed through, an article. The authors have granted ASM a non-exclusive, world-wide license to publish the Supplemental Material files. Please contact the corresponding author directly for reuse.
