# Supplementary material for: Δlpp mutant E. coli reduces lipid content in Caenorhabditis elegans via phosphatidylglycerol-mediated inhibition of fatty acid biosynthesis
Source: mSystems. 2026 Jun 17;11(7):e00155-26. doi: 10.1128/msystems.00155-26 (PMC13386898; doi:10.1128/msystems.00155-26)
Supplement: Supplemental text — Extended methods. [file msystems.00155-26-s0001.docx]

**Methods**

***C. elegans* culture conditions**

*C. elegans* (N2, LIU1 and LIU2) were purchased from the Caenorhabditis Genetics Center (USA). *dhs-25, acs-5, acs-15, acs-18* mutants were purchased from the National BioResource Project. *C. elegans* strains were maintained at 20 °C on *E. coli* using standard cultivation techniques ^1^. *C. elegans* were maintained on high-fat NGM medium (1.5 g NaCl, 1.25 g peptone and 8.5 g agar in 475 mL deionized water, with 0.5 mL CaCl2, 0.5 mL MgSO4, 0.5 ml K3PO4 and 0.5 mL cholesterol solution (5 mg/mL in ethanol)) and fed with *E. coli* BW25113 as a food source. *E. coli* BW25113, and *Δlpp* mutant were purchased from Dharmacon (USA).

**Lipid visualization by oil red O staining**

Oil Red O staining to detect the lipid content was performed as previously described ^2^. In short, collect synchronous worms at 60 hours of age, wash three times with M9 buffer, then fix with 500 μL of 4% paraformaldehyde for 30 minutes, freeze and thaw three times at -80 °C, discard the supernatant and wash three times with M9 buffer. At room temperature, the worms were stained with an oil red O staining solution consisting of 60% oil red O stock solution and 40% water for 15 minutes. Remove the dye and wash three times with M9 buffer solution. Finally, the stained worms were mounted and imaged using a fluorescence microscope Nikon DS Ril, Nikon Corporation, Tokyo, Japan. The Image J software V1.8.0.112 was used for quantitative analysis of density. At least 30 nematodes were checked each time, and the process was repeated three times independently.

**RNA extraction and whole transcriptome RNA resequencing**

RNA was extracted from *C. elegans* samples using the TransZol UP Plus Kit. After passing the quality control via electrophoresis, the extracted *C. elegans* RNA underwent purification, rRNA removal, fragmentation, cDNA synthesis, and end repair processes using the RNA Clean XP Kit and RNase-Free DNase Set. The concentration of the constructed libraries was measured using a Qubit2.0 Fluorometer, and their size was detected with an Agilent2000. Finally, the libraries were sequenced on an Illumina NovaSeq6000 sequencer in PE150 mode. Applying Seqtk (https://github.com/lh3/seqtk) to filter Reads to obtain clean Reads. The split mapping algorithm of Hisat2 was used to perform Genome mapping on preprocessed reads ^3^. Count the number of Fragments for each gene after Hisat2 alignment using Stringtie^4^. Calculate the TPM value of each gene using RNAnorm.

**Lipidomics analysis**

Lipidomics analysis was performed at Majorbio, China, on a platform consisting of an independent ultra-high performance liquid chromatography-tandem mass spectrometry (UPLC-MS/MS) instrument. The mass spectrometric data were collected using a Thermo UHPLC-Q Exactive HF-X Mass Spectrometer equipped with an electrospray ionization (ESI) source operating in positive mode and negative mode. The raw data from the mass spectrometry instrument was imported into LipidSearch (Thermo, CA) for peak detection, alignment and identification. The preprocessing results generated a data matrix that consisted of the lipid class, retention time (RT), mass-to-charge ratio (m/z) values, and peak intensity.

**Calculating pathway activity score based on RNA-seq data**

Single-sample gene set enrichment analysis (ssGSEA) is a method used to evaluate the activity on a certain gene set or pathway for a sample. We obtained the lipid-related pathways gene sets from the KEGG database. Based on RNA-seq data from *C. elegans*, ssGSEA scores were calculated using the GSVA R package ^5^. The lipid-related pathways gene sets pathways were performed differential analysis by student’s t test.

**Gene ontology annotation and pathway enrichment**

DEGs discovered in this study were annotated using the DAVID database ^6^. For GO annotation, we chose significantly enriched GO terms (p-value < 0.05) from biological process (BP) as the result. For pathway enrichment, we chose significantly enriched KEGG pathways and Reactome pathways (p-value < 0.05) as the result. Data visualization was performed using ImageGP 2^7^.

**Bioinformatics analysis**

Genes with absolute value of logFC > 1 and FDR < 0.05 were considered as significantly differentially expressed genes using DESeq2^8^. We used the multivariate analysis OPLS-DA model, combined with p-values by student’s t test, to screen differentially expressed metabolites with VIP > 1 and FDR < 0.05. The significant differences in nematode staining intensity were completed using the student’s t test. The significant difference in ssGSEA scores between two groups was completed using student's t-test. The STORMS Checklist for standardize reporting of human microbiome research has been uploaded to zenodo (https://zenodo.org/records/18768322)^9^.

**References**

1 Stiernagle, T. Maintenance of C. elegans. *WormBook*, 1-11, doi:10.1895/wormbook.1.101.1 (2006).

2 Xu, A. *et al.* Microtubule regulators act in the nervous system to modulate fat metabolism and longevity through DAF-16 in C. elegans. *Aging Cell* **18**, e12884, doi:10.1111/acel.12884 (2019).

3 Kim, D., Paggi, J. M., Park, C., Bennett, C. & Salzberg, S. L. Graph-based genome alignment and genotyping with HISAT2 and HISAT-genotype. *Nat Biotechnol* **37**, 907-915, doi:10.1038/s41587-019-0201-4 (2019).

4 Li, B. & Dewey, C. N. RSEM: accurate transcript quantification from RNA-Seq data with or without a reference genome. *BMC Bioinformatics* **12**, 323, doi:10.1186/1471-2105-12-323 (2011).

5 Hanzelmann, S., Castelo, R. & Guinney, J. GSVA: gene set variation analysis for microarray and RNA-seq data. *BMC Bioinformatics* **14**, 7, doi:10.1186/1471-2105-14-7 (2013).

6 Sherman, B. T. *et al.* DAVID: a web server for functional enrichment analysis and functional annotation of gene lists (2021 update). *Nucleic Acids Res* **50**, W216-W221, doi:10.1093/nar/gkac194 (2022).

7 Chen, T. *et al.* ImageGP 2 for enhanced data visualization and reproducible analysis in biomedical research. *Imeta* **3**, e239, doi:10.1002/imt2.239 (2024).

8 Love, M. I., Huber, W. & Anders, S. Moderated estimation of fold change and dispersion for RNA-seq data with DESeq2. *Genome Biol* **15**, 550, doi:10.1186/s13059-014-0550-8 (2014).

9 Mirzayi, C. *et al.* Reporting guidelines for human microbiome research: the STORMS checklist. *Nat Med* **27**, 1885-1892, doi:10.1038/s41591-021-01552-x (2021).
