# Supplementary material for: Δlpp mutant E. coli reduces lipid content in Caenorhabditis elegans via phosphatidylglycerol-mediated inhibition of fatty acid biosynthesis
Source: mSystems. 2026 Jun 17;11(7):e00155-26. doi: 10.1128/msystems.00155-26 (PMC13386898; doi:10.1128/msystems.00155-26)
Supplement: Supplemental text — Fig. S1 to S7. [file msystems.00155-26-s0002.docx]

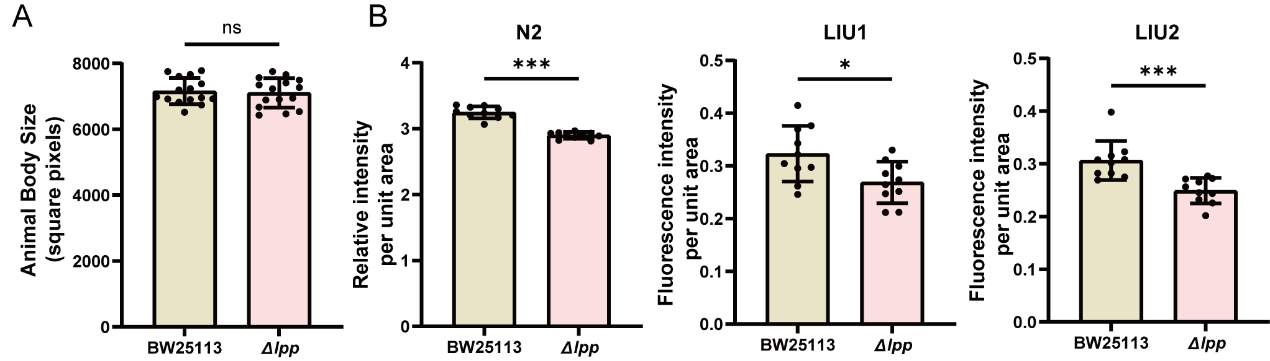


Supplementary Figure 1 The effect of feeding *Δlpp* mutant on the body size, relative intensity of oil red and Fluorescence intensity per unit surface area of nematodes.


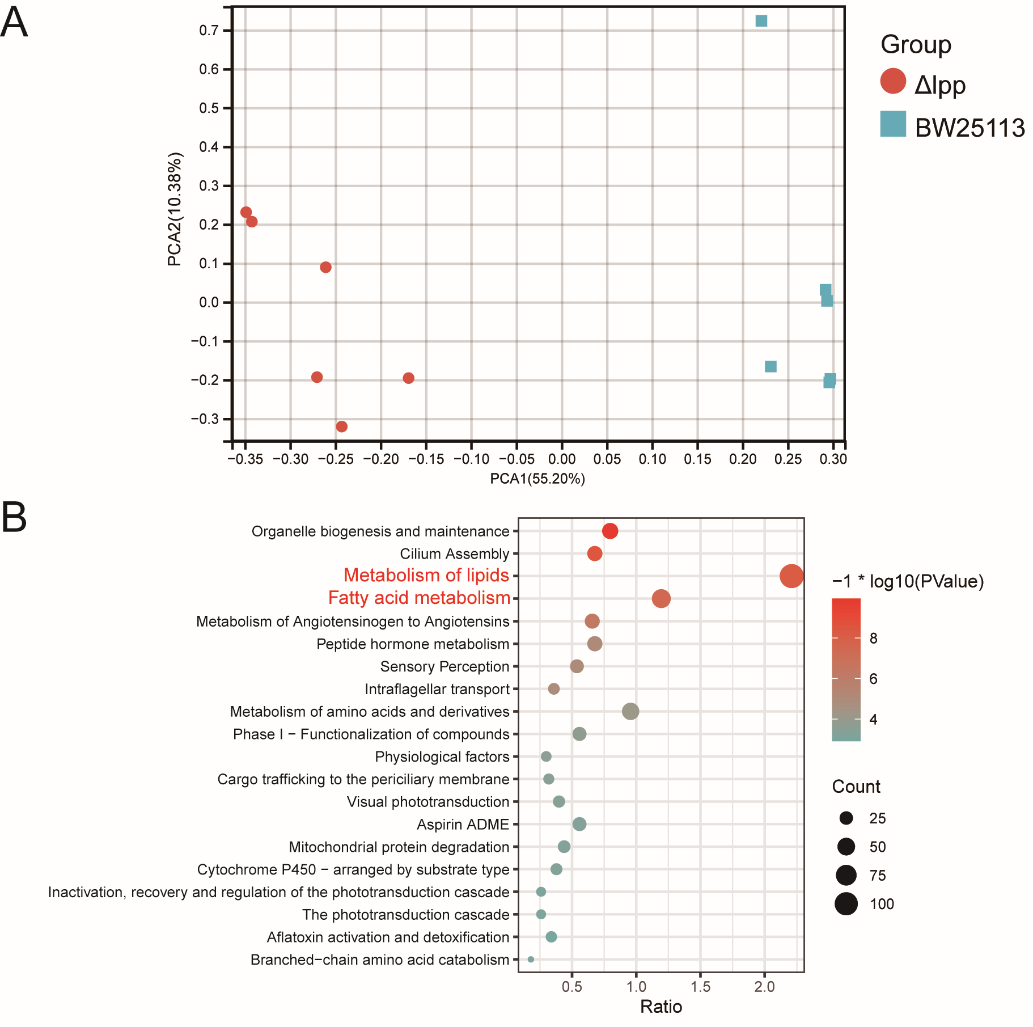


Supplementary Figure 2 (A) Principal components analysis (PCA) based on gene expression profile. (B) Reactome Pathway enrichment analysis of differentially expressed genes.


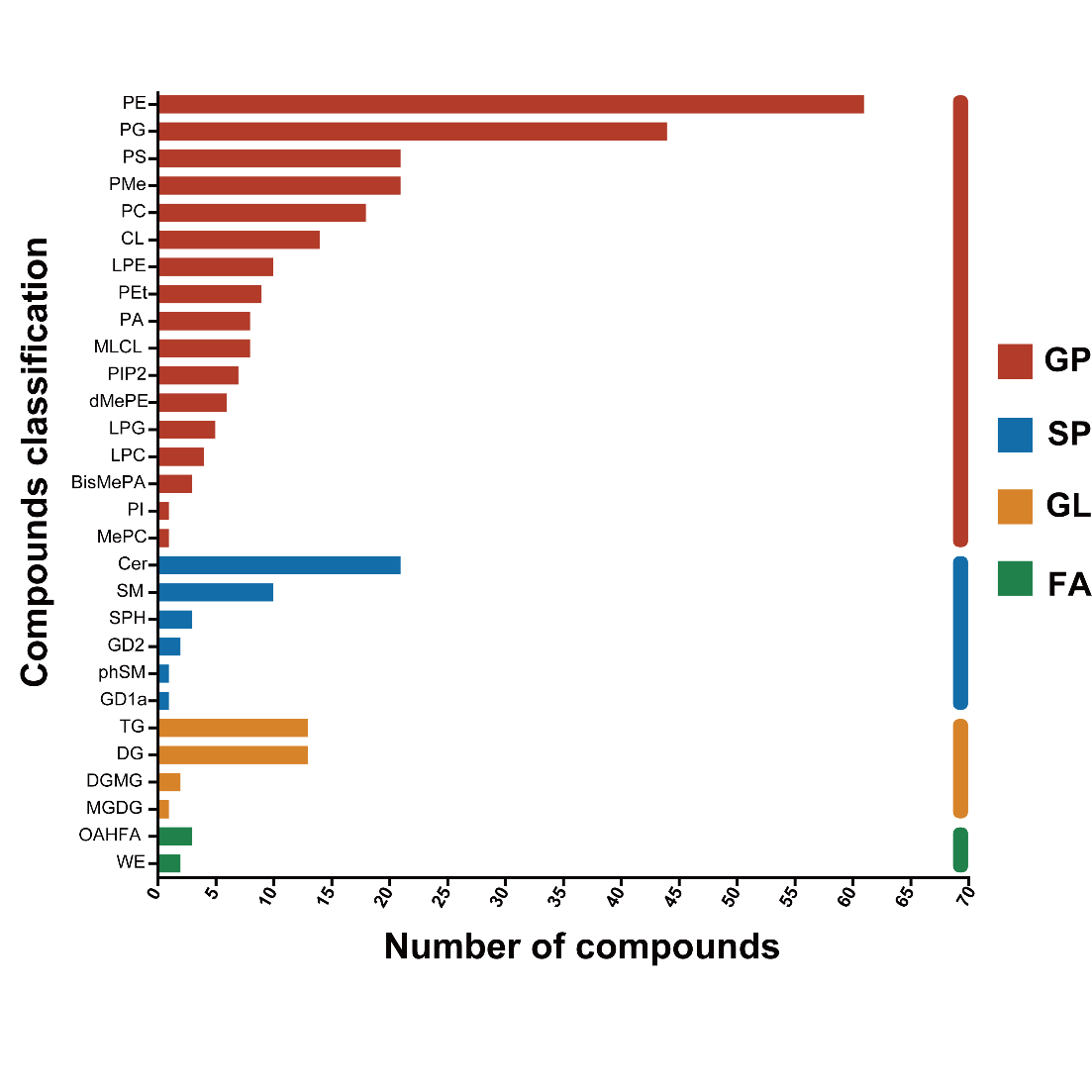


Supplementary Figure 3 All lipid metabolites detected by the LC-MS analysis platform and their corresponding classifications.


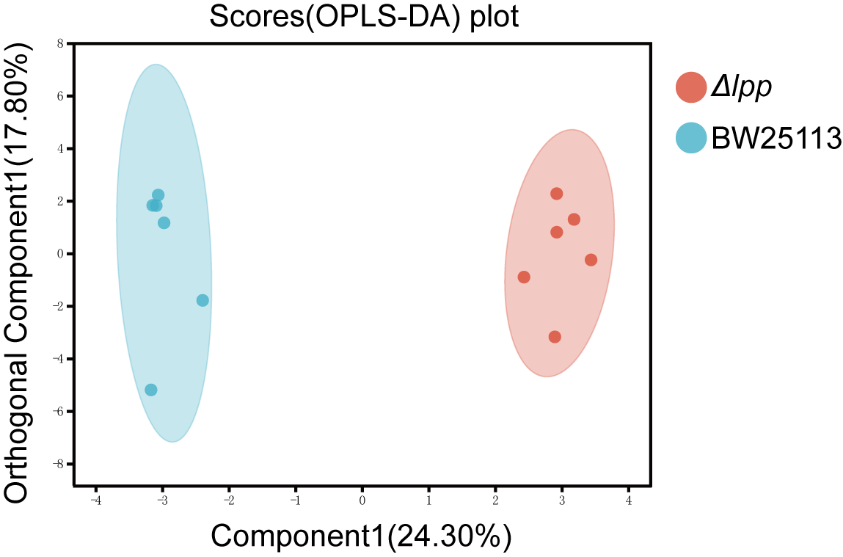


Supplementary Figure 4 OPLS-DA analysis of samples from *Δlpp* and BW25113groups based on lipid metabolic profiling.


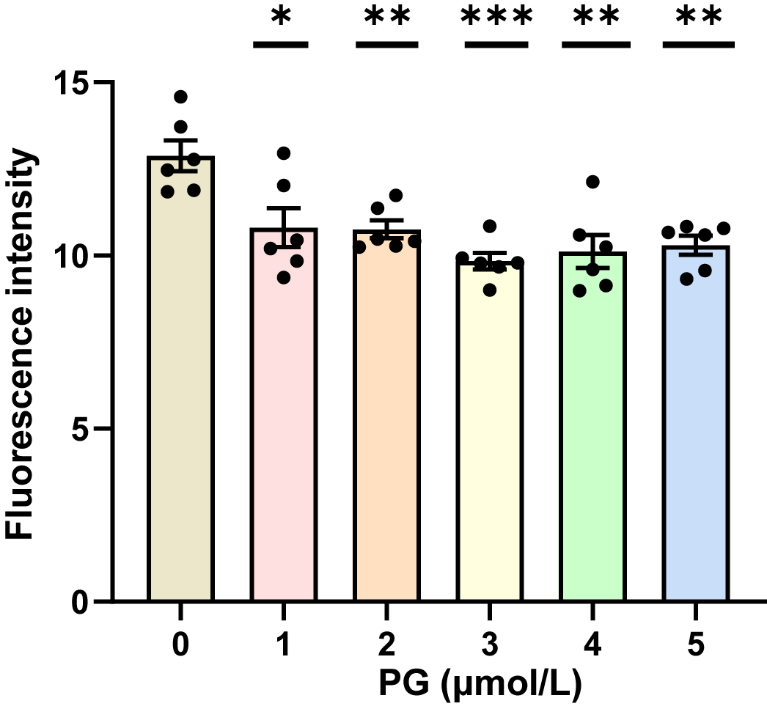


Supplementary Figure 5 Among all tested concentrations, 3 μM PG caused the greatest reduction (14.8%, p < 0.001) in fluorescent intensity of lipid content in LIU1 nematodes.


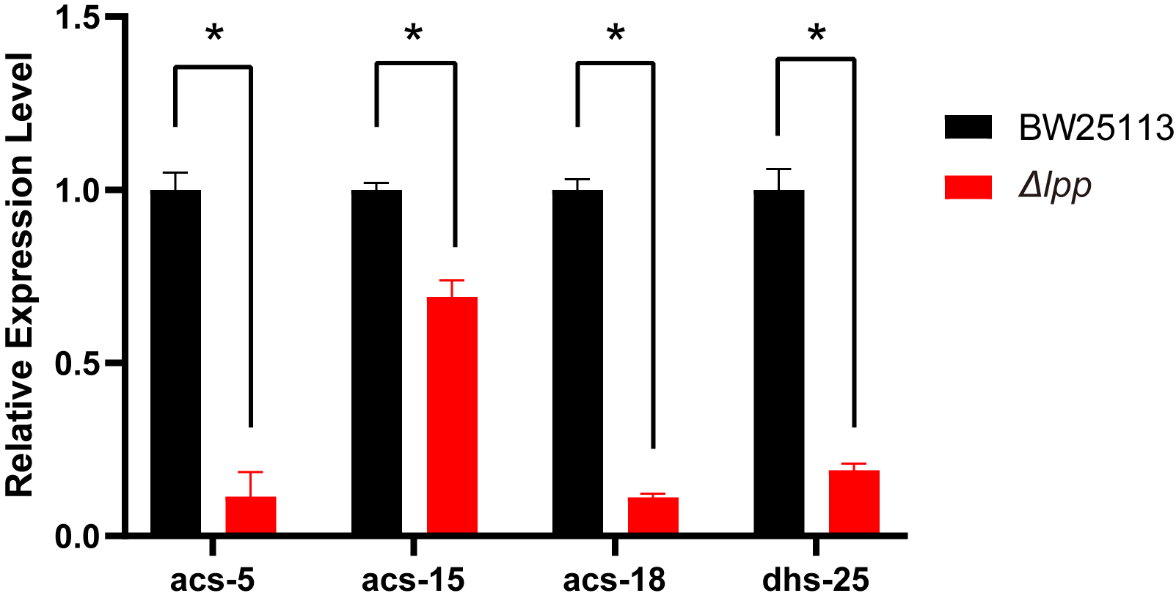


Supplementary Figure 6 Assessment of four key genes expression levels via qPCR in *Δlpp* group and BW25113 group.


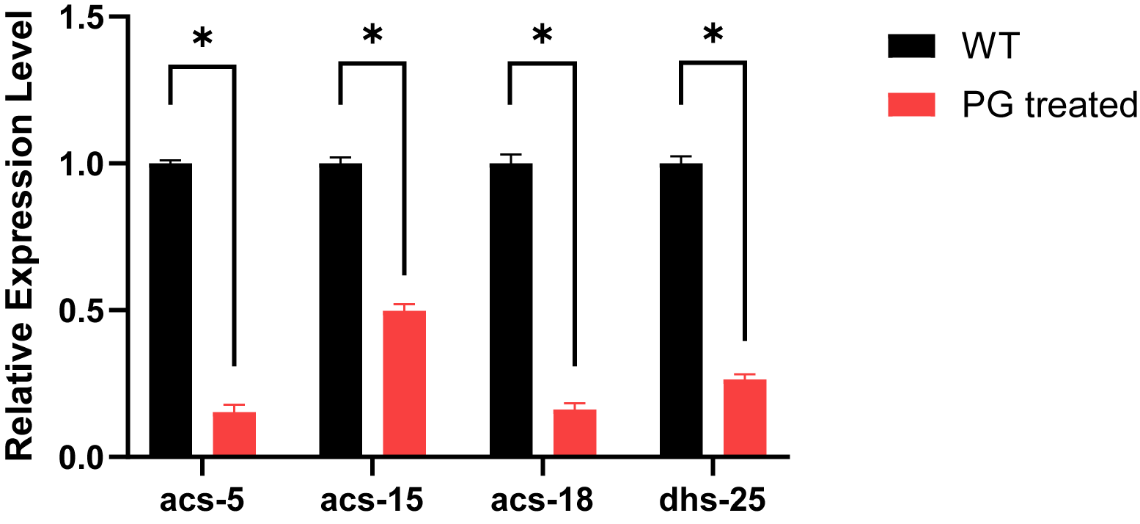


Supplementary Figure 7 Assessment of four key genes expression levels via qPCR in PG-treated group and WT group.
