# Supplementary material for: Δlpp mutant E. coli reduces lipid content in Caenorhabditis elegans via phosphatidylglycerol-mediated inhibition of fatty acid biosynthesis
Source: mSystems. 2026 Jun 17;11(7):e00155-26. doi: 10.1128/msystems.00155-26 (PMC13386898; doi:10.1128/msystems.00155-26)
Supplement: Reviewer comments [file reviewer-comments.pdf]

AMERICAN SOCIETY FOR MICROBIOLOGY | mSystems®

Δlpp Mutant *E. coli* Reduces Lipid Content in *Caenorhabditis elegans* via Phosphatidylglycerol-Mediated Inhibition of Fatty Acid Biosynthesis

Dianshuang Zhou, Liwei Tang, Mengdie Ni, Wen Yang, Xiangming Wang, Ying Li, and Zuobin Zhu

Corresponding Author(s): Zuobin Zhu, Xuzhou Medical University

Review Timeline:

Submission Date:	February 26, 2026
Editorial Decision:	March 19, 2026
Revision Received:	April 24, 2026
Accepted:	May 12, 2026

Editor: Marnix Medema

Reviewer(s): Disclosure of reviewer identity is with reference to reviewer comments included in decision letter(s). The following individuals involved in review of your submission have agreed to reveal their identity: Daoming Wang (Reviewer #2)

Transaction Report:

(Note: With the exception of the correction of typographical or spelling errors that could be a source of ambiguity, letters and reports are not edited. The original formatting of letters and referee reports may not be reflected in this compilation.)

DOI: https://doi.org/10.1128/msystems.00155-26

Re: mSystems00155-26 (***Δlpp* Mutant *E. coli* Reduces Lipid Content in *Caenorhabditis elegans* via Phosphatidylglycerol-Mediated Inhibition of Fatty Acid Biosynthesis**)

Dear Prof. Zuobin Zhu:

Thank you for the privilege of reviewing your work. Below you will find my comments, instructions from the mSystems editorial office, and the reviewer comments.

Thank you for your patience in waiting for the reviews of your manuscript. From their comments, it is clear that your revision has clearly improved the paper. Yet, as you will see, one of the reviewers still raises important points that would need to be addressed carefully before we can consider accepting the article for publication. I do expect that these are addressable, mostly by textual changes, and that we will be able to accept a version of the manuscript in which you e.g. clarify the screening process and present the mechanistic conclusions more cautiously.

Please return the manuscript within 60 days; if you cannot complete the modification within this time period, please contact me. If you do not wish to modify the manuscript and prefer to submit it to another journal, notify me immediately so that the manuscript may be formally withdrawn from consideration by mSystems.

Revision Guidelines
To submit your modified manuscript, log into the submission site at https://msystems.msubmit.net/cgi-bin/main.plex. Go to Author Tasks and click the appropriate manuscript title to begin. The information you entered when you first submitted the paper will be displayed; update this as necessary. Note the following requirements:

• Upload point-by-point responses to the issues raised by the reviewers in a file named "Response to Reviewers," NOT in your cover letter.
• Upload a compare copy of the manuscript (without figures) as a "Marked-Up Manuscript" file.
• Upload a clean .DOC/.DOCX version of the revised manuscript and remove the previous version.
• Each figure must be uploaded as a separate, editable, high-resolution file (TIFF or EPS preferred), and any multipanel figures must be assembled into one file.
• Any supplemental material intended for posting by ASM should be uploaded with their legends separate from the main manuscript. You can combine all supplemental material into one file (preferred) or split it into a maximum of 10 files with all associated legends included.

For complete guidelines on revision requirements, see our Submission and Review Process webpage. Submission of a paper that does not conform to guidelines may delay acceptance of your manuscript.

Data availability: ASM policy requires that data be available to the public upon online posting of the article, so please verify all links to sequence records, if present, and make sure that each number retrieves the full record of the data. If a new accession number is not linked or a link is broken, provide mSystems production staff with the correct URL for the record. If the accession numbers for new data are not publicly accessible before the expected online posting of the article, publication may be delayed; please contact production staff (mSystems@asmusa.org) immediately with the expected release date.

Publication Fees: For information on publication fees and which article types are subject to charges, visit our website. If your manuscript is accepted for publication and any fees apply, you will be contacted separately about payment during the production process; please follow the instructions in that e-mail. Arrangements for payment must be made before your article is published.

ASM Membership: Corresponding authors may join or renew ASM membership to obtain discounts on publication fees. Need to upgrade your membership level? Please contact Customer Service at Service@asmusa.org.

The ASM Journals program strives for constant improvement in our submission and publication process. Please tell us how we can improve your experience by taking this quick Author Survey.

Thank you for submitting your paper to mSystems.

Sincerely,
Marnix Medema
Editor
mSystems

Reviewer #1 (Comments for the Author):

The author's response has been fully addressed my concerns. The quality of the paper has apparently improved. I agree with the publication of this article.

Reviewer #2 (Comments for the Author):

This study combines in vivo phenotyping, transcriptomics, and bacterial lipidomics to investigate how a specific E. coli mutant (Δlpp) influences host lipid metabolism in C. elegans, and identifies phosphatidylglycerol (PG) as a candidate mediator. The multi-omics design and the integration of complementary experimental approaches are strengths of the work, and the data consistently support that increased PG exposure is associated with reduced lipid accumulation in the host.

However, several conceptual issues remain insufficiently resolved. In particular, the current framing tends to overstate the mechanistic role of lpp deletion, while the data more directly support a PG-driven effect. The distinction between metabolite-mediated effects and gene-specific mechanisms requires clearer articulation. In addition, the rationale and transparency of the mutant screening strategy need to be substantially improved. Finally, parts of the introduction would benefit from more accurate positioning within the existing literature and improved narrative clarity.

Specific comments
- The current results clearly demonstrate that increased PG exposure is sufficient to reduce lipid accumulation in C. elegans. However, this raises an important interpretational issue. The observed phenotype may primarily reflect a change in the metabolic input provided by the bacteria (i.e., increased PG availability), rather than a specific regulatory mechanism associated with lpp deletion.
In this sense, Δlpp may function mainly as a perturbation that alters bacterial PG supplement, analogous to changing the nutrient content of the diet, rather than revealing a distinct genetic mechanism. Consistent with this interpretation, the data suggest that any perturbation that increases PG availability or intake could potentially lead to a similar lipid-lowering effect. The current framing therefore appears to conflate the effect of a metabolite (PG) with the effect of a specific genetic perturbation (lpp deletion).
I recommend that the authors clarify this distinction and present their conclusions more cautiously, as the data more directly support a PG-driven effect than a lpp-specific mechanistic pathway.

- The authors have attempted to address my previous comment by adding a small number of recent references. However, the core statement that "knowledge about the influence of genetic variation in gut microbiota on host health remains limited" is still overly general and potentially misleading. A substantial body of literature has already demonstrated that microbial genetic variation-including strain-level diversity, gene content variation, and structural variation-can influence host physiology and disease phenotypes. Simply adding a few citations does not resolve this issue, as the current wording still overlooks the maturity of this research area. More importantly, the statement lacks specificity. It remains unclear in what sense the knowledge is "limited"-whether in terms of causal inference, mechanistic resolution, or specific experimental systems. I recommend that the authors substantially revise this part of the introduction by:
(1) providing a more balanced and accurate overview of existing work on microbial genetic variation (such as gene presence/absence, copy number variation, structural variation, SNPs, and strain/lineage...) and host phenotypes;
(2) clearly defining the specific gap that this study addresses (e.g., single-gene perturbation, metabolite-mediated mechanisms, etc.), rather than using a generic statement.
Without this clarification, the motivation of the study remains conceptually weak.

- The rationale and procedure used to identify Δlpp as the focal mutant remain insufficiently described. The authors state that they "systematically screened mutant genes related to lipid metabolism from the KEGG database," yet the criteria used to define lipid metabolism-related genes are not specified. It is unclear whether this classification was based on KEGG pathways, functional annotations, or manual curation. In addition, the scale and design of the screening process are not described: the total number of candidate genes, whether all candidates were experimentally tested (e.g., via knockout and Oil Red O staining), and, if not, how Δlpp was prioritized over other candidates all remain unclear. This lack of transparency makes it difficult to determine whether Δlpp emerged from a systematic and unbiased screen or from a more selective exploration.
Furthermore, it is not evident how lpp fits within the stated category of lipid metabolism genes. Based on KEGG annotation (e.g., K06078), lpp encodes a major outer membrane lipoprotein involved in cell envelope structure rather than a canonical lipid metabolism enzyme, and it is not directly assigned to core lipid metabolic pathways such as fatty acid biosynthesis or glycerophospholipid metabolism. This apparent inconsistency between the stated screening strategy and the functional annotation of lpp further complicates the interpretation. The authors should therefore clarify how lipid metabolism-related genes were defined, whether lpp was included based on specific or indirect criteria, or whether its identification arose from a broader screening framework. If the latter is the case, the description of the screening process should be revised accordingly to avoid potential misinterpretation.?
- While the authors have added a description of lpp function later in the manuscript (around line 173), the gene is already mentioned much earlier (around line 65) without sufficient context or explanation. At this point in the text, lpp appears abruptly, with no prior description of its biological role or relevance, which may be confusing for readers who are not already familiar with this gene. This disrupts the logical flow of the introduction and weakens the clarity of the study's rationale. I recommend that the authors provide a brief functional introduction of lpp at its first occurrence (following the last comment, after the systemic

screening would be much smooth), or alternatively restructure the text so that its biological role is introduced before it is discussed in the context of the experimental findings.

- Related to my previous comment ("What is the impact of lpp deletion on the bacterium?"), the current response does not adequately address the intended question. My concern specifically refers to the effect of lpp deletion on the bacterium itself, rather than on host phenotypes. Given that lpp encodes a major outer membrane lipoprotein involved in cell envelope structure, its deletion is expected to influence bacterial properties such as membrane integrity or cell morphology.
However, the data provided by the authors primarily describe host lipid metabolism in C. elegans, and do not clarify how the Δlpp mutation affects E. coli itself under standard growth conditions (e.g., in vitro, outside the host). I recommend that the authors clarify what is known about the phenotypic consequences of lpp deletion in E. coli, either by providing relevant experimental data or by discussing established findings from the literature. This would help better contextualize the observed effects and strengthen the mechanistic interpretation of the study.

- Figure 2F, the color should also be consistent with the colors used in Figure 1.

Reviewer #1 (Comments for the Author):

The author's response has been fully addressed my concerns. The quality of the paper has apparently improved. I agree with the publication of this article.

Reply:

Dear Reviewer

Thank you for your recognition of the revisions to this paper. We are very pleased to receive your affirmation. We sincerely appreciate your valuable suggestions, which have played a crucial role in improving the quality of the paper.

Sincerely,

Zuobin Zhu

Reviewer #2 (Comments for the Author):

This study combines in vivo phenotyping, transcriptomics, and bacterial lipidomics to investigate how a specific E. coli mutant (Δ*lpp*) influences host lipid metabolism in C. elegans, and identifies phosphatidylglycerol (PG) as a candidate mediator. The multi-omics design and the integration of complementary experimental approaches are strengths of the work, and the data consistently support that increased PG exposure is associated with reduced lipid accumulation in the host.

However, several conceptual issues remain insufficiently resolved. In particular, the current framing tends to overstate the mechanistic role of *lpp* deletion, while the data more directly support a PG-driven effect. The distinction between metabolite-mediated effects and gene-specific mechanisms requires clearer articulation. In addition, the rationale and transparency of the mutant screening strategy need to be substantially improved. Finally, parts of the introduction would benefit from more accurate positioning within the existing literature and improved narrative clarity.

Reply:

Dear Reviewer

We greatly appreciate your careful review of this paper and your valuable constructive comments. We fully agree with the points you have raised and will revise the relevant content accordingly in the revised manuscript. All revisions have been marked in the marked-up manuscript.

We hope the revised manuscript will meet your requirements. Below you will find our point-by-point responses to the comments and questions.

Sincerely,

Zuobin Zhu

1. The current results clearly demonstrate that increased PG exposure is sufficient to reduce lipid accumulation in C. elegans. However, this raises an important interpretational issue. The observed phenotype may primarily reflect a change in the metabolic input provided by the bacteria (i.e., increased PG availability), rather than a specific regulatory mechanism associated with *lpp* deletion.

In this sense, Δ*lpp* may function mainly as a perturbation that alters bacterial PG supplement, analogous to changing the nutrient content of the diet, rather than revealing a distinct genetic mechanism. Consistent with this interpretation, the data suggest that any perturbation that increases PG availability or intake could potentially lead to a similar lipid-lowering effect. The current framing therefore appears to conflate the effect of a metabolite (PG) with the effect of a specific genetic perturbation (*lpp* deletion).

I recommend that the authors clarify this distinction and present their conclusions more cautiously, as the data more directly support a PG-driven effect than a *lpp*-specific mechanistic pathway.

Reply 1: We are very grateful for your profound insights into this manuscript. It is important to distinguish between the effects of PG metabolites and the gene-specific mechanism of *lpp* deletion for an accurate interpretation of the findings in this study. We agree that our data most directly support a PG-driven effect, and we will clarify this interpretation in the revised manuscript。We will clearly state that the primary discovery is that increased availability of bacterial PG (whether via Δ*lpp* mutation or direct supplementation) is sufficient to reduce host lipid accumulation.

We have modified our text as advised:

This study found that the *Δlpp* mutant inhibits fatty acid biosynthesis and fat accumulation in the host by increasing the production of PG and suggests that regulating the export of specific metabolites by the gut microbiota may represent a potential strategy for modulating host metabolism.

2. The authors have attempted to address my previous comment by adding a small

number of recent references. However, the core statement that "knowledge about the influence of genetic variation in gut microbiota on host health remains limited" is still overly general and potentially misleading. A substantial body of literature has already demonstrated that microbial genetic variation-including strain-level diversity, gene content variation, and structural variation-can influence host physiology and disease phenotypes. Simply adding a few citations does not resolve this issue, as the current wording still overlooks the maturity of this research area. More importantly, the statement lacks specificity. It remains unclear in what sense the knowledge is "limited"-whether in terms of causal inference, mechanistic resolution, or specific experimental systems. I recommend that the authors substantially revise this part of the introduction by:

(1) providing a more balanced and accurate overview of existing work on microbial genetic variation (such as gene presence/absence, copy number variation, structural variation, SNPs, and strain/lineage...) and host phenotypes;

(2) clearly defining the specific gap that this study addresses (e.g., single-gene perturbation, metabolite-mediated mechanisms, etc.), rather than using a generic statement.

Without this clarification, the motivation of the study remains conceptually weak.

Reply 2: We are very grateful for your profound insights into this manuscript. Our previous statements were too general and did not accurately reflect the progress in this field. We have now completely rewritten the relevant sections in the introduction to address this issue. Specifically, we have restructured the narrative by first acknowledging the established body of work linking microbial genetic variations to host phenotypes, thereby providing a more balanced overview. We then briefly summarize our work, which connects the identified bacterial gene perturbations to metabolite-mediated reductions in lipid content. This clarification reinforces the conceptual rationale for using single-gene knockout models to dissect this precise “gene-to-metabolite-to-phenotype” relationship.

We have modified our text as advised:

Previous studies have demonstrated that genetic variations in microorganisms,

including gene presence/absence, structural variations, and copy number alterations, are closely associated with host physiology and disease phenotypes. Obesity is associated with a higher copy number of thioredoxin 1 (K03671) in the genus Clostridium[9]. A recent study further reveals that bacterial structural variations, particularly those involved in ion and amino acid metabolism as well as bacterial growth regulation, are significantly associated with autism spectrum disorder and hold promise as diagnostic biomarkers[10]. Our previous research demonstrated that *S. cerevisiae* mutant strains can regulate the lifespan, sleep patterns, and locomotor performance of *Drosophila melanogaster* [11]. In this study, we found that the *Δlpp* mutant *E. coli*, which lacks a gene encoding the major outer membrane lipoprotein *Lpp* (a key structural component covalently linking the outer membrane to peptidoglycan)[12], significantly reduced the lipid content in *C. elegans*, and its metabolite PG was a key molecule for this effect.

3. The rationale and procedure used to identify Δ*lpp* as the focal mutant remain insufficiently described. The authors state that they "systematically screened mutant genes related to lipid metabolism from the KEGG database," yet the criteria used to define lipid metabolism-related genes are not specified. It is unclear whether this classification was based on KEGG pathways, functional annotations, or manual curation. In addition, the scale and design of the screening process are not described: the total number of candidate genes, whether all candidates were experimentally tested (e.g., via knockout and Oil Red O staining), and, if not, how Δ*lpp* was prioritized over other candidates all remain unclear. This lack of transparency makes it difficult to determine whether Δ*lpp* emerged from a systematic and unbiased screen or from a more selective exploration.

Furthermore, it is not evident how *lpp* fits within the stated category of lipid metabolism genes. Based on KEGG annotation (e.g., K06078), *lpp* encodes a major outer membrane lipoprotein involved in cell envelope structure rather than a canonical lipid metabolism enzyme, and it is not directly assigned to core lipid metabolic pathways such as fatty acid biosynthesis or glycerophospholipid metabolism. This apparent inconsistency

between the stated screening strategy and the functional annotation of *lpp* further complicates the interpretation. The authors should therefore clarify how lipid metabolism-related genes were defined, whether *lpp* was included based on specific or indirect criteria, or whether its identification arose from a broader screening framework. If the latter is the case, the description of the screening process should be revised accordingly to avoid potential misinterpretation?

Reply 3: We greatly appreciate your detailed and insightful comments, which are crucial for improving the transparency and rigor of our methodological section. The original description contained ambiguities regarding the screening criteria, scale, and workflow, which could lead to misunderstanding. In response to each of your points, we will clarify and revise the manuscript as follows.

Identification of Candidate Genes:

Step 1: Genes involved in all lipid metabolism–related pathways were extracted from the KEGG database.

Step 2: Considering crosstalk between pathways, genes exhibiting high-confidence direct physical or functional interactions with the above pathway-derived genes (Score > 900) were retrieved from the STRING database, to capture regulatory factors that may indirectly affect lipid metabolism.

Step 3: By matching against our Escherichia coli genome-wide knockout library, 133 mutant strains with corresponding gene disruptions were obtained.

Screening Method:

These mutant strains were individually fed to Caenorhabditis elegans strain N2, with the wild-type strain BW25113 as the control. Primary screening was performed using Oil Red O staining, with a significance threshold of $P < 0.05$, and each experiment was repeated in triplicate. 44 candidate mutants were identified in the primary screen and further validated in two C. elegans strains LIU1 and LIU2 carrying fluorescent lipid droplet markers. After comprehensively evaluating the lipid-lowering effects of each mutant across the three C. elegans strains, the Δ*lpp* mutant was ultimately selected for subsequent studies.

We have removed vague expressions such as “systematically screened mutant genes related to the lipid metabolism function” and replace them with more precise descriptions. The revised content is as follows:

Based on the KEGG database, functional pathways associated with lipid metabolism were screened, and genes within these pathways were extracted. Genes interacting with those in lipid metabolism–related pathways (interaction score > 900) were obtained using the STRING database. By matching against the *E.coli* genome-wide knockout library, 133 mutant strains with corresponding gene disruptions were acquired. These mutant strains were individually fed to *C.elegans* strain N2, with the wild-type strain BW25113 as the control. Primary screening was performed via Oil Red O staining, with $P < 0.05$ set as the significance threshold, and each experiment was repeated three times. 44 candidate mutants were identified from the primary screen and further validated in two *C. elegans* strains, LIU1 and LIU2, which carry fluorescent lipid droplet markers. After comprehensively evaluating the lipid-lowering effects of each mutant across the three *C. elegans* strains, the Δ*lpp* mutant was ultimately selected for subsequent studies.

4. While the authors have added a description of *lpp* function later in the manuscript (around line 173), the gene is already mentioned much earlier (around line 65) without sufficient context or explanation. At this point in the text, *lpp* appears abruptly, with no prior description of its biological role or relevance, which may be confusing for readers who are not already familiar with this gene. This disrupts the logical flow of the introduction and weakens the clarity of the study's rationale. I recommend that the authors provide a brief functional introduction of *lpp* at its first occurrence (following the last comment, after the systemic screening would be much smooth), or alternatively restructure the text so that its biological role is introduced before it is discussed in the context of the experimental findings.

Reply 4: We are very grateful for your profound insights into this manuscript. We have added a brief background introduction for the *lpp* gene and its function at the first mention of the Δ*lpp* mutant (near line 65, where it states “In this study, we found that the Δ*lpp* mutant *E. coli*…”). Your suggestion has helped ensure the logical fluency of

the manuscript and laid a solid foundation for readers to understand the subsequent research motivation and results.

We have modified our text as advised:

In this study, we found that the *Δlpp* mutant *E. coli*, which lacks a gene encoding the major outer membrane lipoprotein *Lpp* (a key structural component covalently linking the outer membrane to peptidoglycan)[12], significantly reduced the lipid content in *C. elegans*, and its metabolite PG was a key molecule for this effect.

5. Related to my previous comment ("What is the impact of *lpp* deletion on the bacterium?"), the current response does not adequately address the intended question. My concern specifically refers to the effect of *lpp* deletion on the bacterium itself, rather than on host phenotypes. Given that *lpp* encodes a major outer membrane lipoprotein involved in cell envelope structure, its deletion is expected to influence bacterial properties such as membrane integrity or cell morphology.

However, the data provided by the authors primarily describe host lipid metabolism in C. elegans, and do not clarify how the Δ*lpp* mutation affects E. coli itself under standard growth conditions (e.g., in vitro, outside the host). I recommend that the authors clarify what is known about the phenotypic consequences of *lpp* deletion in E. coli, either by providing relevant experimental data or by discussing established findings from the literature. This would help better contextualize the observed effects and strengthen the mechanistic interpretation of the study.

Reply 5: Thank you very much for your continued attention to this critical issue. We also agree that clarifying the known effects of the Δ*lpp* mutation on bacteria themselves is essential for interpreting the subsequent findings of this study. Relevant elaboration have added in the paragraph of the Discussion section. Specifically, we have cited existing literature and note that:

(1) Δ*lpp* mutation leads to significant impairment of outer membrane integrity in Escherichia coli;

(2) Although Δ*lpp* mutation do not affect the fine surface structure of bacteria, they significantly alter cell height, with varying degrees of change;

(3) Although Δ*lpp* mutation does not affect basic growth, it results in markedly increased outer membrane permeability in E. coli.

In this study, the Δ*lpp* mutation caused a significant upregulation of phosphatidylglycerol (PG) levels, which most likely arises from bacterial cell envelope stress and adaptive remodeling triggered by *lpp* deficiency. Such changes in metabolite levels indirectly induced by the deletion of a structural gene provide a material basis for subsequent effects on the host.

We have modified our text as advised:

Braun's lipoprotein encoded by the *lpp* gene is the sole molecule that covalently links the outer membrane to peptidoglycan in *E. coli*[20]. Its deletion compromises outer membrane integrity and increases cell membrane permeability[21,22]. Although *lpp* deficiency does not significant affect the basic growth of *E. coli*[21], outer membrane stress may trigger the reprogramming of bacterial membrane lipid metabolism. In the present study, the *Δlpp* mutation resulted in a significant upregulation of PG levels, which most likely arises from bacterial cell envelope stress and adaptive remodeling induced by *lpp* deletion.

6. Figure 2F, the color should also be consistent with the colors used in Figure 1.

Reply 6: Thank you very much for pointing out that the color of Figure 2F is inconsistent with that of Figure 1. This is crucial for improving the consistency of the figures. We have adjusted the color of Figure 2F. The revised Figure 2F is as follows:

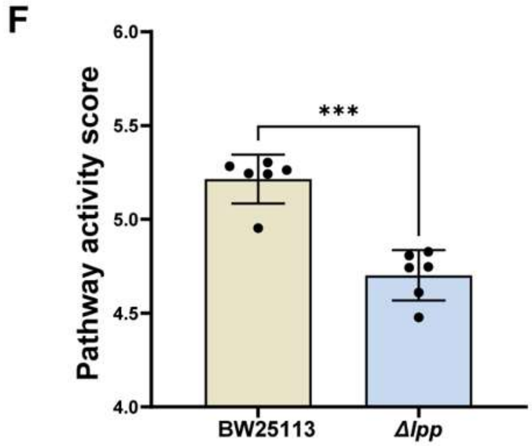

Re: mSystems00155-26R1 (***Δlpp*** **Mutant *E. coli* Reduces Lipid Content in *Caenorhabditis elegans* via Phosphatidylglycerol-Mediated Inhibition of Fatty Acid Biosynthesis**)

Dear Prof. Zuobin Zhu:

Your manuscript has been accepted, and I am forwarding it to the ASM production staff for publication. Your paper will first be checked to make sure all elements meet the technical requirements. ASM staff will contact you if anything needs to be revised before copyediting and production can begin. Otherwise, you will be notified when your proofs are ready to be viewed.

Data Availability: ASM policy requires that data be available to the public upon online posting of the article, so please verify all links to sequence records, if present, and make sure that each number retrieves the full record of the data. If a new accession number is not linked or a link is broken, provide production staff with the correct URL for the record. If the accession numbers for new data are not publicly accessible before the expected online posting of the article, publication may be delayed; please contact ASM production staff immediately with the expected release date.

Specifically, in your case, it seems the RNAseq data still needs to be uploaded to a public repository, and the data availability statement updated accordingly, before we can proceed with processing your article.

Publication Fees: For information on publication fees and which article types have charges, please visit our website. We have partnered with Copyright Clearance Center (CCC) to collect author charges. If fees apply to your paper, you will receive a message from no-reply@copyright.com with further instructions. For questions related to paying charges through RightsLink, please contact CCC at ASM_Support@copyright.com or toll free at +1-877-622-5543. CCC makes every attempt to respond to all emails within 24 hours.

ASM Membership: Corresponding authors may join or renew ASM membership to obtain discounts on publication fees. Need to upgrade your membership level? Please contact Customer Service at Service@asmusa.org.

Embargo Policy: A press release may be issued as soon as the manuscript is posted on the mSystems Latest Articles webpage. The corresponding author will receive an email with the subject line "ASM Journals Author Services Notification" when the article is available online.

Cover Image Submissions: If you would like to submit a potential Cover Image, please email a file and a short legend to msystems@asmusa.org. Please note that we can only consider images that (i) the authors created or own and (ii) have not been previously published. By submitting, you agree that the image can be used under the same terms as the published article. Image File requirements: TIF/EPS, 7.5 inches wide by 8.25 inches tall (at least 2,250 pixels wide by 2,475 pixels tall), minimum 300 dpi resolution (600 dpi preferred), RGB, and no figure elements, e.g., arrows or panel labels. The legend should be a short description of the image, 1-2 sentences recommended. Please download and use this interactive template in Adobe to ensure that your proposed cover image meets our size requirements (https://journals.asm.org/pb-assets/pdf-text-excel-files/ASM-Interactive-Sizing-Cover-Template-1715689791.pdf).

Thank you for submitting your paper to mSystems.

Sincerely,
Marnix Medema
Editor
mSystems

Reviewer #2 (Comments for the Author):

The authors have adequately addressed my previous comments and revised the manuscript accordingly. I have no further comments.
